# Thoughts and experiences on leg amputation among patients with diabetic foot ulcers

**DOI:** 10.1080/17482631.2021.2009202

**Published:** 2021-12-29

**Authors:** Marie Kragh Nielsen, Heidi Bergenholtz, Ulla Riis Madsen

**Affiliations:** a Cand Scient San., Ortopedic Department, Herlev Hospital, Herlev, Denmark; b Surgical Department Holbaek Hospital, Holbæk, Denmark; c Ortopedic Department, Holbaek Hospital, Denmark, Holbæk, Denmark

**Keywords:** Lower limb amputation, patient perspective, perceived self-efficacy, psycho-social consequences, taboo

## Abstract

**Background:**

Although leg amputation is common among patients with diabetic foot ulcers, only few studies have examined the thoughts regarding leg amputation from the perspective of patients.

**Aim:**

This study aims to explore the thoughts of patients with diabetic foot ulcers regarding leg amputation.

**Method:**

A qualitative design using semi-structured interviews were used and analysed using Interpretative Phenomenological Analysis (IPA). In all five patients participated and the interview questions were focused on thoughts in relation to a possible leg amputation.

**Findings:**

Four significant themes were revealed: 1) “Considered—not spoken”—reflections on being alone with one’s thoughts, 2) “What people think about me”—concerns about consequences on social relations, 3) “The tough ones and the ones who whine”—considerations about expected self-efficacy and 4) “Limitations and opportunities”—thoughts about physical consequences.

**Conclusion:**

Even if an amputation is not yet planned, having a diabetic foot ulcer can result in divergent thoughts regarding leg amputation. The findings indicate that amputation is considered a taboo which makes it difficult for the patient to talk about it within either the health care context or with relatives. Health care professionals should therefore be aware of how they communicate regarding leg amputation.

## Background

Treatment of a diabetic foot ulcer is complex, long-lasting and involves multiple actors (Barg et al., 2017; Bus et al., 2020; Madsen, 2017; Sundhedsstyrelsen, 2011). Ten percent of persons with a diabetic foot ulcer will have leg amputation (Jeffcoate et al., 2006) and it is evident that leg amputation is a major life-event that influences all aspects of life (Cornell & Meyr, 2018; Madsen, 2017; Wang et al., 2018). However, there is growing awareness that having a leg amputation after a long and devastating trajectory of wound care could also result in increased quality of life (Barg et al., 2017; Madsen, 2017), and patients with diabetic foot ulcers have reported that they have got their life back after amputation (Madsen, 2017). A study by Dillon et al (Dillon et al., 2020) indicates that patients demand better information on the prognosis and consequences of possible amputation. Psychiatrists have reported that patients expect negative social attention after a leg amputation. Specifically, there is fear among these patients that the psychosocial aspects of life after an amputation will affect them as much as the physical aspects (Bhuvaneswar et al., 2007). This is based on the clinicians’ perspectives and only few qualitative studies have investigated the expected consequences of diabetic foot ulcers and amputations from the perspective of the patients (Kuhnke et al., 2014).

The thoughts of patients and their relatives while considering major leg amputation have been investigated by Wang et al (Wang et al., 2018). This study revealed that all participants had concerns about survival, being ill and the experienced consequences of having treatment for foot ulcers. Concerns about the necessity of the amputation were discussed, and consulting to get a second opinion was common (7. This in accordance with a study by Barg et al (Barg et al., 2017) which addressed the fact that thoughts about having an amputation were often seen as something to be feared. In particular, loss of independence was a major concern (Barg et al., 2017). To gain insight into the perceived concerns of patients at risk of lower extremity amputation because of unspecified foot ulcer, Cornell and Meyr (Cornell & Meyr, 2018) found that patients were concerned about becoming dependent and not regaining walking ability. They also found less frequent concerns included pain, footwear, economic effect and physical appearance (Cornell & Meyr, 2018).

As described only few studies have investigated the thoughts of patients with foot ulcers regarding leg amputation from their perspective. Therefore, the aim of this study was to explore the thoughts of patients with diabetic foot ulcers in relation to leg amputation.

## Method

### Design

**Figure 1.**
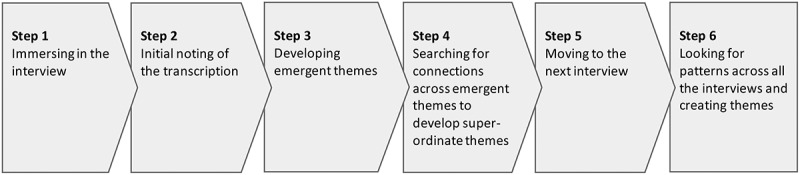
Analysis by IPA

A qualitative interview study design was used, and the analysis was guided by the Interpretive Phenomenological Analysis (IPA) designed by Smith, Flowers and Larkin (Smith et al., 2009). IPA is based on an ideographic approach and the purpose of IPA is to gain insight into people’s experiences from the participant’s perspective. The overall method for IPA, is illustrated in Figure 1.

### Setting

Participants were recruited from four outpatient wound clinics in Denmark, three in an urban area and one in a rural. All clinics had a multidisciplinary team specialized in diabetic wound care.

### Sample

The study was carried out from January-June 2019 by the first author. Criteria for inclusion were Danish speaking, age > 18 and having current diabetic foot ulcer treatment in an outpatient wound clinic. A convenience sample (Creswell & Plano Clark, 2007) of five participants was included. A sample size up to six participants, are recommended for IPA studies (Smith et al., 2009) as a relatively small group of participants included accommodates depth of analysis. Eligible patients were identified by wound nurses who provided an information leaflet and obtained verbal permission for the first author to contact the patients by phone. Nurses were concerned that patients who did not know the association between diabetic foot ulcers and amputation might be worried by being interviewed on this topic. To address this concern, patients they knew had this knowledge were recruited. Patients were excluded if they had had or planned to have an amputation at any level or if they had dementia. Four men and one woman participated. The ages of the participants ranged from 40 to 80 years, and the participants had experienced diabetic foot ulcers between three months to five years prior to the interviews. Participants were no longer in the labour market, and four lived alone (Table I). All but one participant knew someone (family, friend or acquaintance) who had had leg amputation.

**Table I.** Characteristics of participants. Names are psuedonyms

Participant	Sex	Age	Civil status	Wound history	Diabetes
Morten	male	46	living alone	Current wound < 1 year. Had a wound for > 4 years in the past	type 2 > 10 years
Gert	male	53	living alone	>2 years	type 2 > 5 years
Steen	male	72	living alone	>1 year	type 2 < 1 year
Niels	male	73	married	< ½ year	type 2 > 10 years
Bente	female	70	living alone	< ½ year	type 2 > 10 years

### The process of data generation

Data was generated through semi structured interviews undertaken by the first author and performed at the participants’ homes. The interviews followed a thematically designed interview guide (appendix A) with open-ended questions (Kvale & Brinkmann, 2015). The interview guide addressed possible thoughts, considerations and reflections in relation to physical, mental and social conditions, related to a leg amputation. Questions were tested for relevance and appropriateness by an experienced wound nurse and was pilot tested in the first interview, without giving rise to any changes. Furthermore, were all participants at the end of the interview asked: “Is there anything important about the topic of leg amputation that you think we have not been around? ”. Additional questions and probes were asked when appropriate, and interviews were initiated with a brief introduction intended to define the subsequent interview situation. This was followed by background questions (Kvale & Brinkmann, 2015). A broad introductory question—“Can you tell me what you know about leg amputation?”—was asked as broad initial questions can result in spontaneous answers and thus provide insight into what the participant finds important regarding a given phenomenon (Kvale & Brinkmann, 2015). Interviews lasted between 32–80 minutes and were digitally recorded and transcribed verbatim by the first author.

Analysis using an IPA approach means that it is the researcher’s task to interpret the participant’s narrative and shape it into an analytical narrative that is supported by concrete words and phrases from the participant (Smith et al., 2009). As set forth by IPA, each interview used in the current study was analysed separately before the next participant interview. This was done by carefully listening, transcribing, and reading through the transcription (step 1). Notes were then written next to the transcriptions (step 2) and emergent themes and super-ordinate themes were created from what was gleaned from the notes (step 3–4). When all interviews had been analysed, the super-ordinate themes were then compared, and themes describing patterns across interviews were constructed (step 6) (Smith et al., 2009). Table II illustrates examples of the process of analysis.

**Table II.** Three examples of work with empirics in step two, three, four and six of IPA. Names are pseudonyms

Notes	Transcription	Emergent theme	Super-ordinate theme	Themes based on patterns across the interviews
Morten compares death and leg amputation. Where do such comparison come from? Taboo—for whom and why? What is it about leg amputation that is so terrible?	Niels: *“[…] Well, it´s like you´re not talking about death … it´s like … a little taboo-like”*	Comparison between death and leg amputation	Leg amputation—a taboo	Considered—not spoken
Gert has tried to talk to his friends about his foot ulcer and his own thoughts on potential leg amputation, but his friends dont´t want to talk about it. Reluctance? Why is it so difficult to talk about? Why don´t his friends want to talk about it, even though he brings up the topic himself?	Gert: *“[…] here after I got my foot ulcer, I´ve mentioned it sometimes, but it´s not something people [his friends] want to talk about.”*	Leg amputation—difficult to talk to others about.	Other people´s view on legamputation	
Morten considers leg amputation as punishment because he has not taken care of his diabetes. Punishment. But who is punishing and why? Who decides if you have looked after your diabetes well enough? Whose responsibility is it? The health services, individual or in combination?	Morten: *“Well, I could see it as some kind of punishment because I didn´t take good care of myself.”*	Responsibility for health and illness.	View on own situation	

The first author was responsible for data generation and analysis. Two senior researchers participated in the process of analysis by reading all coded text, discussing the emergent themes, and collaborating in writing up the manuscript. The first author, being a novice researcher, had continuous reflections on her own role as an interviewer which she recorded by writing down personal preconceptions, keeping a logbook and dialoguing with the other authors. An example of a preconception that was reflected on, was the following description of a meeting with a participant: “The participant appeared in worn clothes and bare feet. In addition, the home appeared a bit cluttered and dirty, and there was, among other things, a large hole in some furniture that looked like it had been caused of a blow. These impressions gave rise to prejudice in me that the participant probably would not be able to express particularly reflective thoughts, and reflections. This prejudice was challenged throughout the interview and turned out to be wrong. The participant contributed with very rich empirics.”

## Ethics

Principles for research given in the Helsinki Declaration and the Northern Nurses’ Federation were followed (World Medical Association, n.d.). Participants gave informed written consent to be part of the study. According to Danish law, formal ethical approval of the study was not required (Datatilsynet, Justitsministeriet, 2018). As described earlier, participants were selected from those whom nurses knew were informed about the association between wounds and the risk of amputation. However, being interviewed on sensitive topics can evoke strong emotions. Therefore, the interview ended with a debriefing and agreements were made with the referring nurses that the participants could contact them if needed.

## Findings

The themes identified in the analysis were: 1) “Considered—not spoken”—reflections on being alone with one’s thoughts, 2) “What people think about me”—concerns about consequences in social relations, 3) “The tough ones and the ones who whine”—considerations on expected self-efficacy, and 4) “Limitations and opportunities”—thoughts about physical consequences.

Considered—not spoken. Reflections on being alone with one’s thoughts

The participants were aware that their foot ulcers could lead to leg amputation. They also had the perception—and some the experience—that leg amputation is something you don’t talk about. After the interviews, several participants said that it had been pleasant but unfamiliar to put their thoughts into words. They described leg amputation as a topic “*similar to death*”, a “*hush-hush-topic*” and “*taboo-like*”. One participant regarded leg amputation as a self-inflicted punishment due to inadequate self-care and said:

I know that I’m at risk of a leg amputation […] and well, I could see it as some kind of punishment because I haven’t taken proper care of myself. Now that punishment comes bit by bit. (male, 46 years, >four years of wound experience)

He recounted that on “*bad days”* he had mentioned amputation as a solution for his troublesome foot ulcer to healthcare professionals in the outpatient wound clinic but that they had talked him out of it. This participant also mentioned how his family had laughed at him and dismissed it when he had mentioned leg amputation. He did not feel his family accommodated and supported him, and he had therefore not mentioned it again. Another participant had experienced that his friends would not talk about leg amputation. He said:

I think it’s because they don’t want to hear it themselves … they don’t like it, it’s ugly […] I don’t know if it’s because people think it’s disgusting … I think they only see it in horror movies where they chop off the legs of people. It’s the only amputation you see and hear people talk about, right? (male, 53 years, two years of wound experience)

When asked what important knowledge they would like to have prior to leg amputation, several participants spontaneously replied that they would like to speak with someone in the same situation. They all mentioned self-help groups as in other contexts (e.g., cancer). Four of them imagined that meeting with like-minded people would provide concrete answers to questions such as what the experience of collaboration with the municipality had been like, whether the amputation site itched afterwards and the reactions of others to the amputation. It was stated that this kind of meeting could provide hope and an opportunity for open discussion. One participant did not want to use a self-help group himself because he reasoned that he was from a generation where you take care of your own problems.

What people think about me—concerns about consequences in social relations

The participants expected positive reactions from their personal network if leg amputation became relevant. But there were worries and fears that the encounters with strangers would be a challenge because the participants were sure they would be viewed negatively. One described it as follows:

My family and friends, they know me, and they take me as I am. It’s probably more strangers on the street. I know how it is. You can stand and point fingers at people. (male, 53 years, two years of wound experience)

Encounters with strangers was described as one of the things that would affect them the most after a leg amputation. One mentioned leg amputation as a manifestation of illness and described what would be at the top of his mind this way;

The mental distress of what people would think about one. You know … when you suddenly have to consider it. (male, 46 years, >four years of wound experience)

All participants wanted to use a prosthesis to regain mobility and independence if a leg amputation became a reality. However, several also stated that they would hide the prosthesis when encountering strangers in order to hide the amputation and thus avoid negative attention. One described the use of prosthesis as follows:

[…] so you can … look normal, right. […] Well, it would mean that maybe I could walk around … Uh … well, so it seems real … well, it’s … then you normalize yourself, even though you are missing a leg. (male, 53 years, two years of wound experience)

Another said:

I think if you are wearing long pants and they can see that you are limping, then I don’t think they will look at you in the same way as if you came with a leg prosthesis [that could be seen]. (male, 46 years, >four years of wound experience)

The tough ones and the ones who whine—considerations on expected self-efficacy

Some participants had difficulties imagining their expected self-efficacy after a leg amputation while others had a variety of different thoughts ranging from fear to faith. Getting a leg amputated was described as a crossroad where you could be brave or lose courage. One participant described it as:

Some will probably be tough … and avoid pity from others … others will probably sit in a chair. And I probably belong to that category myself, who will sit and say, ‘God, it’s such a pity for me’. Right […] they will probably sit and whine. (male, 46 years, >four years of wound experience)

On the other hand, another participant had the belief that he would be able to cope with a leg amputation and would regard it as a chance for survival. He said:

A tree would also be cropped if a branch has withered. That’s how it is. Otherwise it will not survive. (male, 53 years, two years of wound experience)

A third participant was considering whether a leg amputation could be a positive thing as he found that the foot ulcer inhibited him and prevented him from going outside:

I could look on the bright side that I would be able to go outside. That I don’t have to just walk in here with both feet. (male, 73 years, < six months of wound experience)

Several of the participants referred to how they had previously handled health issues and, based on these experiences, described what mattered for them: encouragement from others, knowing other amputees and humour.

These participants stated that they wanted healthcare professionals to provide them with knowledge—both before and after a leg amputation. One participant described it this way:

[…] they ‘[the medical personnel] should take control ‘[of the dialogue], because you might not be on top of things and tend to be rather indifferent and then you might not even think about it until once you’ve gotten the amputation … then it probably would have been nice to have been prepared beforehand […] that you are sitting with a doctor who you feel will be with you all the way and tell [you] everything. (male, 46 years, >four years of wound experience)

Those who had previously faced difficult health challenges seemed to have greater expectations of their self-efficacy.

### Limitations and opportunities—thoughts about physical consequences

The interviews revealed concern about the limitations an amputation would result in. They also showed that the participants were considering new opportunities. Participants used words such as “*inhibitory*”, “*annoying*” and “*cumbersome*” when describing their expectations of physical ability following a leg amputation. They described a leg amputation as limiting their mobility and resulting in reliance on others although several also had thoughts about whether their foot ulcers might be more inhibitory. Participants were concerned about being a nuisance to their relatives following a leg amputation due to reduced mobility. One said:

It would be stressful for my wife (male, 73 years, < six months of wound experience)

And another:

I think I would be much more dependent on my family and others to help me with various chores […] the less of a burden I am, the better I feel about myself. (male, 46 years, >four years of wound experience)

He also feared a leg amputation could prevent him from being the grandparent he wanted to be. One participant had actually prepared his home for a leg amputation despite of the fact he was not facing amputation. He said:

In fact, I [have] already prepared [for leg amputation] a bit at home if you look (he pointed at the ramps he had added to his home to make mobilization easier) … if I were in a wheelchair. (male, 53 years, two years of wound experience)

All participants considered the use of a prosthesis as an opportunity to regain independence. One had considered whether he would be granted a prosthesis on grounds of his old age. Several participants mentioned phantom pain, and one feared it. This participant believed a professional explanation would make it easier to cope with. Another had the following thoughts:

Since I have no feelings in my legs today, [I think about] whether it [the phantom pain] would be severe or if my brain has already become accustomed to missing a leg? (male, 73 years, < six months of wound experience)

## Discussion

This study shows that even though participants were not currently facing leg amputation, they had many different thoughts regarding leg amputation. For example, they have had thoughts of fear, hopes, worries, reflections and considerations regarding what a leg amputation would mean to them. The findings in this study show that these thoughts were shared by others to a very limited extent. Several participants did not share their thoughts and experience at all with others due to a fundamental belief that leg amputation is taboo and something one does not talk about—not even with health professionals or relatives.

*Tabooization*

This study shows that leg amputation is viewed as a taboo topic that is difficult to discuss. It also illuminates that patients can experience a need to articulate their thoughts on the topic. When people meet others, normative expectations emerge, and deviations may be viewed as taboo and result in stigma (Goffman, 2009). The lack of dialogue concerning amputation, even in interactions with health professionals, could underpin the notion that leg amputation is viewed negatively and is seen as a taboo which could result in stigmatization. Other explanations for not discussing the topic could be fear of what a leg amputation entails or fear of being a nuisance to relatives. A previous study (Barg et al., 2017) describes an initial negative perception of leg amputation as a worse complication to diabetes than death, and it has been found that health professionals viewed and communicated about leg amputation negatively. In another study (Cornell & Meyr, 2018), words such as anxiety and fear were linked to amputation. Thus, several studies (Barg et al., 2017; Bhuvaneswar et al., 2007; Cornell & Meyr, 2018; Dillon et al., 2020; Sanz-Nogués et al., 2020; Wang et al., 2018) substantiate that amputation may be viewed negatively and this may contribute to the tabooization of leg amputation. Our study shows, in accordance to existing findings that diabetic foot ulcers can be the cause of major challenges, but also that the quality of life after a leg amputation can be improved (Barg et al., 2017; Madsen, 2017).

In order to assist with the de-tabooization of talking about leg amputation and to meet any need to articulate thoughts about it, health professionals should be advised to engage in dialogue on this topic—even if the patient is not currently facing amputation. This study underpins previous findings which describe the desire for those who are facing leg amputation to have encounters with like-minded individuals in the same situation (Dillon et al., 2020). It also shows that such encounters can be uplifting (Wang et al., 2018). According to The Canadian-American sociologist Erving Goffmans (1922–1982), the bearer of a stigma will experience acceptance and confidence in meeting others with the same stigma (Goffman, 2009). Findings in the current study can therefore be an expression of a desire for an accepting space where leg amputation can be freely discussed. A Danish national clinical guideline in the field of amputation (Madsen et al., 2020) recommends peer support as it can have a beneficial effect on psychological adaptation after an amputation (Jelic, 2003; Purk, 2004; Richardson et al., 2020). Facilitation of meetings where patients who are going through an amputation can meet with others should therefore be accommodated.

### The importance of physical appearance

Due to the visible disability of leg amputation, this study shows that worries exist about encountering strangers which leads to a desire for the use of a prosthesis. Goffman uses the term *to pass* to describe how a person with a given distinctive character will try to hide it (Goffman, 2009). The use of a covered prosthesis could maintain the illusion of having two legs and meet this normative expectation in society. Previous findings describe expectations of unwanted attention in encounters with a stranger (Barg et al., 2017). These studies have also found that the cosmetic aspect of an amputation can be just as important as the physical aspect for the amputee (Bhuvaneswar et al., 2007). Patients with a high degree of social awareness have been shown to have more difficulty adapting to life after an amputation (2004). This is, however, in conflict with a study by Cornell and Meyr (Cornell & Meyr, 2018) who found that physical appearance was one of the things that participants were least concerned about. It must therefore be assumed that there are different perceptions of the importance of physical appearance which illustrates a need for individual clarification on what thoughts the individual may have on physical appearance after an amputation.

### Self-efficacy

This study shows that there can be very different expectations of self-rated self-efficacy and coping following a leg amputation. These expectations range from overwhelming to manageable. Earlier experiences and the importance of the role of healthcare professionals in providing knowledge and support were emphasized by the participants. According to the Canadian-born psychologist Albert Bandura (1925), self-rated self-efficacy can be influenced by performance accomplishments, vicarious experiences, verbal persuasion, and emotional arousal and is an expression of self-evaluated confidence in dealing with challenging situations (Bandura, 1977; Horgan & Maclachlan, 2004). This was also seen in this study. Understanding the individual’s way of dealing with challenges is important (Bhuvaneswar et al., 2007), and it may be advisable to uncover the patient’s self-rated self-efficacy as a basis for assessing the individual need for support based on the patient’s coping ability.

An Irish study (2015) based on semi-structured interviews of nine men with lower limb amputation due to diabetic foot ulcer emphasized the importance of emotional support, reassurance, and communication. Other studies have also highlighted the importance of empathy, support (Wang et al., 2018), information and education (Dillon et al., 2020). Similar findings have been observed in other patient groups as a study on fast-track total hip and knee arthroplasty in Denmark (2015) described how information and education could contribute to increased empowerment for older patients. Hence, unearthing an individual’s degree of self-efficacy should be considered during the care of diabetic foot ulcers (2020). Healthcare professionals should be aware of their role in providing adequate communication while accommodating, supporting and guiding both the patient and their relatives as they create the best conditions for coping with the new reality.

### Reconciliation of expectations

Leg amputation has been associated with increased dependence. The current study shows that participants were worried about being a burden to their relatives. The study also shows that the use of a prosthesis can be viewed as a realistic opportunity to regain independence. Previous findings similarly described concerns about increased dependence after leg amputation (Barg et al., 2017; 2020; Wang et al., 2018). To address this, it could be advised that dialogue about physical ability, expectations and possible change of roles in the family happen prior to an amputation. In relation to the desire for a prosthesis in Denmark, it is estimated that it is only realistic for 1/3 of the leg amputees to receive a prosthesis (Madsen, 2017) and that the benefit from prosthesis use depends largely on the patient’s physical starting point as well as the level of amputation (Bhuvaneswar et al., 2007). Patients with unrealistic expectations of elective alloplasty surgery may become more realistic regarding their situation through education prior to the surgery (2015). This emphasizes the importance of dialogue on mobility and prosthesis use prior to amputation. This dialogue should include whether this is a realistic goal as well as what could make it realistic.

### Strengths and limitations

The limitation of this study is related to the small group of predominantly male patients. The small sample is a limitation in the transferability of the results but as the aim of using the IPA approach is to gain in-depth insight into the experiences from the participant’s perspective, we judged the size to fit following the recommendation from IPA (Smith et al., 2009). With the use of semi-structured interviews and the IPA analysis, the current study attempts to achieve methodological and theoretical coherence to increase credibility and transparency. This study investigates the thoughts about amputation among patients having diabetic foot ulcers. From this, we cannot conclude whether these thoughts will change, if an amputation becomes a reality.

## Conclusion

The findings from this study show that patients with diabetic foot ulcers may have many unspoken thoughts in relation to leg amputation. This could support healthcare professionals with the insight that patients with diabetic foot ulcers, despite not currently facing leg amputation, may have many thoughts regarding leg amputation and may need help to articulate them.

Moreover, the findings show that leg amputation can be perceived as a taboo topic and therefore difficult to address in a professional healthcare context and with relatives. This knowledge is crucial for the healthcare professionals as they play a significant role in the care of patients, and their work can help contribute to the de-tabooization of the topic.

## APPENDIX A—INTERVIEW GUIDE

**Table:**

**Interview guide**		
I am writing a thesis about what thoughts, reflections and considerations people with a diabetic foot ulcer may have in relation to leg amputation. I have not worked with either people who have had diabetic foot ulcers or amputations and with my thesis I would like to gain insight into some of the thoughts, considerations and reflections that you as a person with diabetic foot ulcers might have about leg amputation. Today it is your thoughts, reflections and considerations that I ask for, so there are no right or wrong answers. I will record our conversation so that I can remember what we have talked about and then write down our conversation verbatim. Parts of the interview I will then use in my thesis and perhaps in a public article but of course your name will not be used. If there is something you would like to say without it being recorded, just say so. The interview will probably last approximately 60 minutes. If you are in doubt about something along the way, need a break or something else then of course just say so. In the interview, I will use the term “leg amputation” and to be sure that we are speaking from the same notion of what that covers. It will be an amputation of the lower leg (I show them my leg). Is there anything you want to ask before we start?		
	**Interview questions**	**Additional questions**
**Background questions**	I just want to start by hearing how old you are?	
	What is your marital status?	
	Do you have any kids?	Possibly grandchildren?
	What is your employment status?	
	How long have you had diabetes?	Type 1 or Type 2?
	How long have you had your ulcer on your foot?	Do you know how your ulcer came about?
		Have you ever had ulcers on your feet that required treatment before?
	Can you tell me what you know about leg amputation?	Why do you think leg amputation is sometimes done?
		How do you know? (Doctor, media, etc.)
	Do you remember if you have ever known or met someone who had a leg amputation?	It could also be someone you had just heard of/seen a random who had a leg amputation?
		Do you remember what thoughts you had when you were told/heard/saw that they had had a leg amputation?
	How do you think you will do after a leg amputation?	
	What are your thoughts about whether you could find yourself in a situation where leg amputation could be a treatment option for your foot ulcer?	
**Possible thoughts, considerations and reflections in relation to physical conditions related to a leg amputation**	What thoughts do you think you would have about your physical state if you were to have a leg amputation?	Are there thoughts that would be more dominant than others?
		Would there be anything that scared you?
		Something that would be good?
		What do you think your everyday life would look like if you had leg amputation?
	Do you think there would be some things that would be especially important to you that would help you gain knowledge regarding your physical state after a leg amputation?	Where do you think this knowledge should come from?
		Could there be something the health care professionals could do to prepare you for the physical challenges? (… e.g., pain, activity, etc.)
**Possible thoughts, considerations and reflections in relation to mental conditions in a leg amputation.**	Can you put how you think you would feel psychologically if you had to have a leg amputation into words?	What thoughts would those be?
		Are there thoughts that would be more dominant than others?
		What do you think those thoughts would do to you?
	Do you think as that there would be any psychological concerns that would be particularly important for you as you gained knowledge about a leg amputation?	Where do you think this knowledge should come from?
		Would there be something you would like your health care professional to do to help you in this regard?
		Who do you think should take the initiative to talk about it?
**Possible thoughts, considerations and reflections in relation to social conditions in a leg amputation.**	Is leg amputation something you talk about?	With whom? Where? Why/Why not?
	What do you think you would feel if people knew you had/would have a leg amputation?	Would you be open about it?
		Why/why not?
		What do you think you would talk about?
		Is there something you wouldn’t talk about?
		Do you think it would make a difference depending on whether it was family who knew about it versus, for example, people on the street who saw it?
	How do you think your family and friends would react if you had a leg amputation?	
**De-briefing** How has it been to talk about leg amputation today? I don’t have any more questions. First of all, I want to thank you for talking to me. I want to hear if you have any questions or if you think there is anything else about leg amputation we haven’t discussed? All conversations can sometimes trigger thoughts—even afterwards. It is important that you know that you are always welcome to contact the ambulatory unit if you need to talk about anything.		
